# Induction of Labor in Uræmia

**Published:** 1874-06

**Authors:** Samuel C. Bussey

**Affiliations:** Washington, D. C.


*THE INDIANA JOURNAL OF MEDICINE*— *January,* 1874:

Artificial Induction of Labor in Uræmia.—*By Samuel C. Bussey, M.D., Washington, D. C.*—" It is conceded that puerperal convulsions are dangerous to the life of the child, and that this mortality is not due simply to the convulsive act is shown by the comparative safety of the child in all those cases of convulsions, when the mother is free from albuminuria. Braun says, 'if the mother dies during pregnancy, under uræmic symptoms, it is almost always a dead child that is brought to light by the abdominal section. If, after numerous uræmic convulsive fits, the child is born alive a large quantity of urea is found in the blood taken from the umbilical cord; but if it be born dead, we can immediately after birth, demonstrate the presence of carbonate of ammonia in the fœtal blood. If it happens that the uræmic symptoms have been entirely removed during pregnancy and delivery, or if the fœtus has been cautiously and in good time removed from the cavity, then the life of the child may be permanently saved, if it be mature.' Thus it appears from these researches, that the fœtal mortality is due to the same cause that produces the eclampsia, and consequently may occur independant of it, as has been frequently observed by Simpson, Araun, Smith, Cahen, Rayer, Bedford and others.'

" So that while depletion of the uterus of an eclampsic patient diminishes vastly the peril of the mother, the danger to the child is but slightly diminished, thus showing that this danger is due to other causes than the convulsive act.

"Prof. Thomas, in 1870, says 'the premature and artificial delivery of a child at the eight-and-a-half month of utero-gestation, by our present methods' is to be 'preferred to delivery by the forceps at tenth menstrual period,' and Barnes of London, (1870) repeats his declaration of 1862, 'that it is just as feasible to make an appointment at any distance from home to carry out at one sitting the induction of labor, as it is to cut for the stone. The operation may be brought entirely within the control of the operator.'"

